# Collectanea Medica, Consisting of Anecdotes, Facts, Extracts, Illustrations, Queries, Suggestions, &c.

**Published:** 1815-06

**Authors:** 


### COLLECTANEA MEDICA,

CONSISTING OF

### ANECDOTES, FACTS, EXTRACTS, ILLUSTRATIONS, QUERIES, SUGGESTIONS, &c.

RELATING TO THE

*History or the Art of Medicine, and the Auxiliary Sciences.*

---

**Official Report** *to the French Institute, by* **Messrs. Vauquelin, Pinel,** *and* **Percy,** *on the Manuscript Work presented to the Institute by* **M. Orfila,** *entitled* "Toxicologie Generale."*

THE work which has been entrusted to us to revise, is comprised in two volumes, each divided into two parts, the first comprehending the mineral poisons, and the second the vegetable and animal poisons.

A complete

---

* Several of M. Orfila's experiments on Poisons have been already communicated to the medical world, through the medium of this

A complete treatise on this interesting subject was a desideratum to science and medical jurisprudence; all those which we previously possessed were either incomplete or inaccurate: it was in vain that we looked into some of them for the best method of ascertaining the nature of poisons; in others there was no description of the organic lesions produced by poisonous substances, while the collection of every peculiar effect exhibited was far from affording adequate materials with which to meet every emergency.

It was necessary, therefore, in order to produce a book on the subject, such as might have been expected from the present state of science, to undertake a series of multiplied and highly delicate experiments: this is precisely what M. Orfila has had the courage to undertake, and he purposes to attain the highest possible degree of perfection.

He first describes with care the physical and sensible characters of poisons in their natural state; he afterwards makes known the chemical properties of these substances, noting very exactly the phenomena which they present on the application of the greatest possible number of re-agents.

But what renders this part of his work more interesting, is, that he details the differences which any given poison, when mixed with alimentary substances of various kinds, exhibits with the same re-agents.

Poisons thus mixing themselves in the stomach and intestines with various liquids, disguise their properties either by combining with or decomposing them. The antecedent researches of the author would have been insufficient for his purpose, if he had not studied, in a particular manner, the modifications which the bile, the saliva, the gastric juice, &c. are capable of imposing upon poisons.

In making these experiments, M. Orfila varied the quantities of poison from a dose so small as to be incapable of producing death, up to a quantity more than sufficient; circumstances which frequently produce differences in the effects occasioned by the reagents.

When the author has well ascertained the characters of the poison in its natural state, and has pursued them through its mixtures or combinations with the alimentary substances, as well as with the humours which are met with in the stomach and intestines, he endeavours to ascertain the manner in which it acts in the animal economy, and the effects which flow from it. This is the physiological part. Sometimes an inspection of the organs affected has

---

this Journal. The formal Report to the Institute, of which we have been favoured with a copy, details the whole of these interesting experiments in a more authentic shape, besides giving them the sanction of the high authority of the names of Vauquelin, Pinel, and Percy.

justified justified his prediction; but frequently, also, the mode of action in most poisons is unknown to us: fortunately this is not the most imperfect branch of toxicology.

That which concerns us all most closely, is the art of preventing, arresting, or remedying, the deleterious effects of poisons: thus M. Orfila has collected, with great care, whatever has been written hitherto on counter poisons; subjected to new trials approved antidotes, and frequently, in discovering their fallacy, he has undertaken new enquiries, which have sometimes been followed with the happiest results.

It was thus that he ascertained that every thing which had been suggested as the counter poison to corrosive sublimate was really useless, and that the true antidote to this dreadful poison was albumen diluted in water. He made this experiment, not only on animals, but he had also an opportunity of verifying it on the human subject.

It was in this way, also, that he ascertained, by repeated experiments, that the antidote of verdigrease is sugar,—a happy discovery, and which certainly could not have been attained by theory.

In the fourth article upon each poison, he traces, in some measure, for the use of police officers, the steps which they ought to take to ascertain the nature of the poisonous substance, whether it has been found in its natural state in any person's house, or has been mixed with foreign bodies; whether it has been vomited, or finally found in the stomach or other organs of an unfortunate person who has died from its effects.

We cannot too highly praise the minute order and precision which the author has observed; and he seems to have felt deeply the serious consequences of an erroneous judgment in such cases.

The first volume of M. Orfila's Toxicology contains four classes of mineral poisons, viz. the mercurial, the arsenical, the antimonial, and the cuprous. The second volume treats of mineral poisons also; and the third of vegetable and animal poisons.

We have no hesitation in saying, that the work is as perfect as the present state of our chemical knowledge will admit; and we can assert, that the individual researches of the author have corrected many old errors, and added a great number of truths highly valuable to medical men, who are frequently called to give their assistance to persons poisoned; to the officers of police, whose business it is to see if there has been really poison employed; and finally to humanity itself. But we ought not to expect that the part which treats of vegetable and animal poisons should be equally complete, because, the nature of these poisons not being so well known as that of the minerals, their way of acting on the organs is, consequently, more obscure, and the methods of remedying their effects much more difficult. It is doing much, however, to have opened the career, to have pointed out the progress and indicated the means of attaining the desired object. Time and experience will, it is hoped, gradually perfect this important branch of toxicology.

The second part of the first volume contains a description of the action produced on the animal economy, by the preparations of tin, zinc, silver, gold, bismuth, the concentrated mineral acids, the caustic alkalies, phosphorus, cantharides, lead, iodine; and an appendix on the counter-poisons of corrosive sublimate, arsenic, and liver of sulphur.

He begins with the chemical and medico-legal part, and afterwards examines the effects of the poisons in a physiological point of view. This last part being almost entirely new, we shall present a succinct extract to the Class.

1. The muriate of tin injected into the veins, to the quantity of three-fourths of a grain, acts promptly on the nervous system, and produces death in ten or twelve hours. Introduced into the stomach, it destroys life, by inflaming and corroding it. Six experiments gave the same results.

2. A concentrated solution of sulphate of zinc acts by stupefying the brain, when it is injected into the veins; when introduced into the stomach, to the amount of an ounce, it produces vomiting only; but, if the œsophagus is tied, the animal dies in two or three days, and the stomach is much inflamed. Six experiments also confirmed these facts.

3. One third of a grain of nitrate of silver, dissolved in two drachms of water, introduced into the circulation, produced death in five or six hours, by acting on the lungs and nervous system. When 36 grains were introduced into the stomach, it was not absorbed, and the animal did not die until the fourth or fifth day, in consequence of the inflammation produced by this caustic. Six experiments gave the same results.

4. Three-fourths of a grain of muriate of gold dissolved in a drachm of water, and injected into the veins, gave death in six or seven hours, by attacking the lungs forcibly. When introduced into the stomach to the amount of twelve grains, the animal died in five or six days, and the stomach was corroded; consequently there had been no absorption. Five experiments supported these facts.

5. The nitrate of bismuth, injected into the veins, has its chief action in the nervous system, and kills animals. When introduced into the stomach, it inflames and corrodes it, acting, at the same time, on the lungs, and destroying life very speedily.

6. Some drops of an acid or alkali, injected into the veins, produce death instantly, by coagulating the blood; the sulphuric acid carbonizes it, as it does in common vessels. Introduced into the stomach, it corrodes and perforates it, and the animals soon die, amidst bloody vomitings, frequently attended with most frightful convulsions.

The coagulation of the blood by the alkalies is remarkable, since they prevent this fluid from coagulating when it is drawn from the human body.

It results from all these facts, that the same poisonous substance can can exert its deadly influence on this or that organ, according to the point with which it has been brought in contact.

7. Ammonia and its sub-carbonate injected into the veins, also coagulate the blood; but they act strangely on the nervous system. When introduced into the stomach, at the dose of a drachm or two, they produce death in a short time, and act upon the brain.

8. The muriate of barytes injected into the veins, introduced into the stomach, or applied externally, kills animals very soon, in the midst of frightful convulsions, acting on the nervous system. Six experiments proved this property. Mr. Brodie had already announced a part of these results.

9. Phosphorus, dissolved in oil, and injected into the veins, produces instant death, by being converted into phosphoric acid, which is exhaled through the nostrils, as M. Majendie had already witnessed.

When introduced into the stomach in small cylinders, it passes into the state of phosphoric acid, which corrodes the texture of this organ, and occasions death in a day or two: the stomach of the animal is found to contain less phosphorus than had been employed.

When the phosphorus is dissolved in oil, before it is given to the animal, it is transformed into phosphoric acid. Life is destroyed in a few hours, and the stomach is full of holes. Six experiments proved this fact.

10. The acetate of lead, when introduced into the stomach to the amount of an ounce and a half, occasions abundant vomitings, and death intervenes ten, twelve, or fifteen hours afterwards. On opening the body, a true inflammation of the parts which comprise the digestive canal is found.

If it be curious to endeavour to ascertain the effects produced in the animal body, by poisonous substances, when introduced either by the blood vessels, or by the mouth, it is still more curious, and certainly more useful, to find out the means of preventing the deleterious effects of these bodies, or at least of arresting their progress, when they have already commenced: this has been enquired into in the medical department of M. Orfila's work.

1. Milk is the true counter-poison of muriate of tin, a substance which frequently falls in the way of individuals. The milk is completely coagulated by this salt; the coagulum contains abundance of oxide of tin, and muriatic acid, and this coagulum is not poisonous. Three experiments confirmed this fact.

2. The muriate of soda is the true counter-poison of the nitrate of silver, since it prevented the corrosive effects of this salt. Two experiments demonstrated this.

3. Calcined magnesia, proposed by Pelletier as the most certain method of arresting the action of the acids, succeeds, in fact, very well; several experiments have demonstrated it, but this remedy must be administered very speedily.

4. The sulphates of soda and magnesia are the true counterpoisons of the salts of lead and barytes. There result, from the reciprocal action of these substances, purging salts, which produce the evacuation of abundance of sulphates of barytes and lead. These antidotes must be employed in great quantities, and at frequent intervals.

M. Orfila discovered that the sulphuret of potass, recommended by Navier to check the effects of the metallic salts, was of no use.

5. The acetic acid is the most efficacious remedy in cases of poisoning by the alkalies. M. Orfila made several experiments to prove this.

6. Iodine, having produced, on dead organic substances, effects very analogous to those exercised by the oxy-muriatic acid, M. Orfila was curious to know what were its effects in the living animal economy. When introduced into the stomach in a small quantity, it acts like a slight stimulant, and produces vomiting. In the dose of a drachm, it constantly kills animals whose œsophagus has been tied, producing ulcerations in the mucous membrane. In the dose of two or three drachms, it acts in the same way on animals whose œsophagus has not been tied, and which are several hours without vomiting. It rarely produces death when it has been administered in the dose of a grain or two, and when the animal rejects it in a short time by vomiting. It never destroys life when applied externally.

It acts upon man as upon dogs. M. Orfila, having once taken two grains of iode, experienced nausea; on another occasion, having taken four grains, he had nausea, with symptoms of choaking, vomiting, and a slight oppression; when he took six grains, he had the same symptoms, besides an acceleration of the pulse, and symptoms of colic.

In an appendix to his work, M. Orfila shews that charcoal is not the counter-poison of corrosive sublimate and arsenious acid (white arsenic), as M. Bertrand had announced; 1st, because animals which took six grains of one or other of these poisons, mixed with four times as much charcoal as M. Bertrand employed, died in a day or two, when the œsophagus was tied, and the stomach was strongly corroded. Now, what constitutes the essence of a counter-poison of corrosive substances is the prevention of corrosion. 2dly, Almost all the animals which took this mixture, and whose œsophagus was not tied, died, after having vomited several times; the stomach was found greatly inflamed.

Two only, out of twenty, of the animals which were subjected to these experiments, escaped, and they instantly vomited the poison enveloped in the charcoal.

In order to prove that the charcoal had acted as an envelope only, six grains of the same poison were given to these two animals, enveloped in clay, which they vomited instantly, and were restored. The water of charcoal is not more efficacious.

In the same appendix, M. Orfila establishes by experiments, 1st, that the sulphuret of potash is an energetic corrosive poison; 2d, that in the dose of half a drachm, it produces death in eighteen or twenty hours, when the œsophagus is tied, by determining inflammation or ulceration of the membranes to the stomach, and acting on the nervous system; 3d, that in the dose of three or four drachms, it kills animals in three or four hours, if they are prevented from vomiting. The author made all his experiments upon dogs.

The researches of which M. Orfila has made up the second part of his work, being of so frequent application, and so immediately useful to the preservation of human life, and to medical jurisprudence; the simple and methodical manner in which the author has treated this interesting subject, together with the pain and disgust attendant upon these kinds of experiments, will, doubtless, determine the Institute to grant him permission to publish his Toxicology with their special approbation; and his zeal will, consequently, be redoubled in the perfection of that department of his experiments which shall include the vegetable and animal poisons.

(Signed) Pinel, Vauquelin, Percy.

The Institute approves of the above Report, and adopts its conclusions. G. Cuvier, Secretary.

---

*On the late Plague at Malta;* by Joseph Skinner, Esq. late Surgeon to the Prisoners of War at Malta.

In what way this terrible scourge was introduced into the island of Malta, is not to the present purpose; but it is to be trusted that for the future, whenever infection shall be known to prevail on ship-board, and to hover about the vicinity of any of the British settlements, there will be but one sentiment relative to the precautionary measures to be adopted. At the time of this visitation Malta was substantially a British colony, although not declared so: the Maltese, however, were not brought into that state which could prevent the spreading of the contagion at the onset. They are a people who, being strongly attached to their native soil, seldom migrate to make observations elsewhere. The history of the plague, as referring to their own island, was too remote to serve them as a guide; and it was not until many of them had been swept away, that they could be made to believe in the real existence of the calamity, which they mistook for an ordinary disease. In the interim, bigotry in all ranks was combined with the cupidity natural to the lower class of the Maltese. The churches were as diligently attended as if no mischief could result from the contact of many individuals; the host was paraded with numerous followers; and visits paid to the infected, without scruple and without hesitation, by their relatives and friends. In this way, and by the concealment of pestilential effects, the disease not only spread through the city of Valetta, but was introduced into several of the *casals* or villages, before the strong hand of power could stay its progress.

When these facts are considered, it will not appear surprising that that this dreadful scourge, which for several months devastated the island of Malta, and was subsequently introduced into Gozo, was principally confined to the indigenous inhabitants of those islands. Few of the Turks or Greeks resident at Valetta, the capital, were attacked, if we except those of the lower class of Greeks, who, in common with the felons, were engaged in the hazardous employments which the exigencies of the occasion required. Other foreigners were equally exempt; and the British peculiarly so. It was indeed a matter of fearful wonder to the Maltese, who regard all protestants as heretics, and therefore the fittest subjects for Almighty vengeance, that of all others they should have been most favoured under this visitation. With the exception of two females, who had been abandoned by the military, and were sent, with the charge of children, to the spot destined to receive the population of the infected quarters of Valetta;—of the wife of a serjeant of artillery who resided at Floriana, when the disease raged with violence in that suburb;—and of the last fatal case at Gozo, which will be touched on hereafter, the writer does not recollect any British who were victims to the disease, the soldiery of one of the military corps excepted. At the breaking out of the plague at Valetta, many of the British families resident there sought refuge in the country. It is highly to the honour of those who remained to face the danger, and who were not rivetted to the spot by public employments, that they were most instrumental in checking the propagation of the contagion, displaying, on every occasion in which they volunteered their services to the government, an heroic courage and a zeal beyond all praise!

Fort Manuel, situated opposite to Valetta, on the other side of the Quarantine creek, was, at the beginning, the receptacle of the sick, who were, together with the remaining tenants of infected houses, poured in from the capital in proportion as the disease gained ground, and before pest-hospitals could be prepared for their reception. Huts were constructed in the sequel, to the end that the entire population of the parts of the capital most infected might be cleared. These were, the Mandragio, a low, obscure, and crowded quarter, chiefly inhabited by market people, and another low spot in the vicinity of Fort St. Elmo. Before these measures could be adopted, the population of the buildings of Fort Manuel became overcharged; and here a particular symptom of plague, which does not appear to have been noticed by any writer on the subject, presented itself. Owing to the stimulating quality of the pestilential virus, a furor, or what may be better termed a *satyriasis*, was induced in both the sexes, which required all the vigilance of the attendants to separate them, and that under the most loathsome circumstances of the disease. It is possible that this fact may lead hereafter to a better knowledge of the specific quality of the contagious matter. Subtle and permeating in its nature, in attacking the vital energies, it produces that peculiar excitement in the system, which has been hitherto almost exclusively clusively confined to the pathology of maniacal cases, and of uterine affections in females.

Several of the Maltese medical practitioners, or, as they style themselves, professors, were selected by the government, and allowed handsome salaries, to administer to the sick who had been removed to Fort Manuel, and to watch over the safety of Valetta, by visiting the houses where a suspicion of infection was entertained; at the same time that the most prudent measures were adopted to prevent the concealment of disease. For this purpose, constant visits were paid in the respective districts into which the city was divided. These districts were separated by barriers, to the end that each of them might be the more closely watched, and all unnecessary intercourse between the inhabitants prevented. The latter were, indeed, with the exception of such as could render themselves useful abroad, confined to their homes, the supplies of provisions being carried round by persons appointed for that purpose. Proclamations were issued, pointing out the precautions they had to observe in receiving these supplies, &c. In short, every salutary and restraining measure which the public safety required, was promptly taken by the Government.

Having adverted to the Maltese practitioners, whose exertions for the relief of their fellow citizens was thus called forth, and liberally recompensed, some account of their practice, of the curative means they employed, may be expected. To come, however, at any precise knowledge of the treatment they pursued, baffled all the ingenuity of inquiry. It is not but that they were sufficiently accomplished, in point of study, for the task they undertook. Several of them may, on the other hand, be cited as highly accomplished in their profession;* but it was not an easy task for them to subdue the constitutional timidity under which they laboured, on the sudden appearance of a contagious distemper with which they were only theoretically acquainted. To follow up any thing like a regular method of cure, required a free and unrestrained communication with the patients under their care, which they could not maintain unless exempted from the panic that was spread around them. History records that on the breaking out of the great plague in London in 1665, among other instances of an heroic contempt of danger—of an ardent zeal in the cause of humanity, which could not be abated by any consideration of personal safety—a Dr. Sayer declared that, braving every risk, he would attend indiscriminately the rich and the poor. He persevered until the last, and escaped the contagion.† Nothing of

* The Maltese public have to regret the loss of Dr. Gravagna, a sensible and judicious practitioner, of very amiable manners. He died in the time of the plague, but it is uncertain whether he fell a victim to the disease.

† Among the precautions with which he armed himself, it was his custom to take a copious draught of Madeira wine on leaving his house, and another on commencing his rounds.

this this sort was to be heard of at Malta: it would in truth appear, that every idea of systematic treatment yielded to the powerful impressions of fear, as well at the onset as when the disease raged with the greatest violence. Even when, towards the close, the symptoms became milder, and the attacks less frequent, the same timid caution—the same dread of approaching the patient, so as to be enabled to direct a particular attention to the symptoms of his case—was to be noticed.*

In aid of the Maltese practitioners, several Turks and Greeks were, at the instance of the Government, sent to Malta from Smyrna. They are said to have been useful, not on account of any particular knowledge they possessed of the management of plague, but because they had been so accustomed to it, and in a manner seasoned, that they braved its attacks in their attendance on the sick.

The attempts at plague inoculation were founded on the commonly received opinion, that those who have had the disease (and it is presumable that this was the case with several, if not with all the above individuals,†) are not again liable to infection. They may be less susceptible than others; but a Frenchman who had been servant in an English family at Valetta, and who, having caught the plague, had been sent to Fort Manuel for cure, had, during his stay there, two subsequent attacks, from the latter of which, although a sharp one, he recovered perfectly.

A Neapolitan physician, who had seen much of the plague in Turkey and Greece, was among the boldest of the practitioners. He did not hesitate to approach the sick, and to treat them with freedom. It was his practice to cauterize the pestilential tumours, and towards the close of the disease, when the inflammatory symptoms had subsided, to make a liberal use of cordials and alexipharmics.

---

* At Valetta, in a part of the fortifications, huts were erected for such of the prisoners of war as had been released on the condition of their undertaking the tasks of sweeping the streets, white-washing infected houses, &c. in the event of their being seized by plague. A suspicious case having occurred among them, the Maltese practitioner on duty kept the respectful distance of sixteen paces from his patient. It is true that he was provided with glasses; but how far, with this interval between the parties, they helped him in his timid inquiries, is uncertain.

† Smyrna is said to be never entirely free from plague, more especially in the district occupied by the Turks. In that city, at Alexandria, and indeed wherever the disease is familiar, those whose temperament has enabled them to resist an attack, or who have escaped the contagion under circumstances of the greatest exposure, are selected to administer to the sick. There may be habits in which there is not the slightest susceptibility to receive the infection, as in the example of the natural small-pox.

It remains now to pay a well-merited compliment to the British medical staff, whose exertions were unfortunately required by the breaking out of the plague in the third garrison battalion, and likewise in De Rolle's regiment, consisting entirely of foreigners.

The military pest-hospital was under the management and direction of Ralph Green, Esq. Inspector-general of Hospitals. The patients had every advantage which could be afforded them by skill and science, aided by that courageous zeal which spurns every idea of personal safety, when an imperious duty is to be performed.* Accordingly, the proportion of recoveries was much greater than that which the Maltese practice has to record. Not any of the attendants took the infection. They were made to bathe, and employed frictions of warm oil, as did also the military on duty. In addition to this latter preservative, recourse was had to cold ablutions of vinegar and water.

With the symptoms by which the plague is characterized, it presents others, in its attack, similar to those of the *endemial causus*, or bilious remitting fever, commonly styled in the Mediterranean the fever of the country. At the commencement it, therefore, requires the same antiphlogistic treatment, by bleeding and other evacuants, to diminish the powerful determination to the brain, the oppression of which is among the earliest of the symptoms, accompanied by stupor and delirium. On the subsidence of the inflammatory symptoms, the case having taken a favourable turn, the method of cure is in either fever the same, independently of what belongs to the tumours and other characteristic signs of plague. The symptoms, as they have latterly presented themselves, and the treatment which appears to have been most successful, are described by Ulstadius, who wrote on plague so far back as the commencement of the sixteenth century;† and by Sennertus, who collected all the authorities up to the middle of the seventeenth.

Several persons at Malta, and among others a British merchant, asserted that they could distinguish a glare, a peculiar wildness of the eyes, before the individual himself in whom it was perceived,

* The following melancholy fact is a proof, among others, that an ardent desire to procure information with a view to benefit mankind, will sometimes carry an individual beyond the prescribed limit of his duty. Dr. Mac Adam, physician to the forces, was sent by the governor of Malta to Gozo, to direct the means to be employed in the case of the plague breaking out among the military stationed there. He was particularly enjoined not to incur any personal risk, his being a task of mere superintendance. His anxiety, however, to acquire a precise knowledge of the nature of the disease, led him to pay frequent visits to the pest-hospital, where at length he caught the infection, and was the last victim of the scourge which so long ravaged the islands of Malta and Gozo.

† His Treatise appeared in 1526.

was sensible of the attack of plague, and while he was still following his usual occupations. The writer had to witness the effect of a sudden attack in a Maltese, who had proceeded, apparently in good health, as far as the Conservatory Square at Valetta, when his progress was in a moment arrested. He had just sufficient strength to maintain himself in an erect posture; but was obliged to be supported on either side when taken to the bier on which he was conveyed to the pest-hospital. His eyes were downcast, his countenance pallid, and there was an expression of anxious terror which sufficiently explained the quality of the attack.

Notwithstanding all that had been advanced in their favour by various writers, and more especially by Messrs. Baldwin and Thornton; and in spite of the example of the oil-carriers in Africa, who are represented as enjoying an immunity from plague; the frictions of warm oil had not, on the breaking out of that disease at Malta, all the credit which an after experience showed them to deserve. This will not appear surprising, when it is considered that several late medical writers have spoken with considerable hesitation on the subject. After the numerous trials made at Valetta, and elsewhere in Malta, these frictions, if properly applied,* may be pronounced to be an almost certain prophylactic. It is a justice due to a very intelligent young man, Mr. Thornton, assistant deputy paymaster to the forces, and nephew to our minister of that name, to state that, with his uncle's book in his hand, he was the first strenuously to recommend their employment, at the breaking out of the contagion. Among those who wrought zealously in this cause, Mr. Matthew Fletcher, a British merchant, was foremost and indefatigable. Whenever a case of plague came to his knowledge, he hastened to the spot, beseeching the inmates of the dwelling, by whom a free communication with the infected individual had been kept up, to have instant recourse to the frictions, and supplying the means where these were deficient. The happy result was, that, on the plague ceasing, he was possessed of a long list of the cases he had recorded, in not one of which a failure was to be found; at the same time that, where this preservative was not employed, it was usual to see the disease spread from one individual to another who herded together, until the whole were swept away. A few instances of the beneficial effects of the oil frictions, as tried at Valetta, will suffice, so as to banish all scepticism on the subject. In a family consisting of seven individuals and a female servant, the father and eldest son, who had both been assiduous in attending the host, and had besides visited a relation

* Every part of the body having been well cleansed with water, or with vinegar and water, the frictions are applied with a sponge, as warm as they can well be borne, the eyes being closed to protect them from irritation. This is repeated at least twice a-week, wearing the same linen between the frictions.

labouring labouring under plague, were attacked, and died pretty nearly at the same time. On the first alarm the frictions were employed by the rest of the family, and all escaped, although they had communicated freely with the unfortunate victims of the disease. A French cutler, who had the same number of children, had married his eldest daughter to a person who took the plague of the next door neighbour, as did likewise the second daughter of the family. The two sickened about the same time, and these also were fatal cases; but the remainder of the family, who had had recourse to the oil frictions as soon as the nature of the attacks was ascertained, escaped, without excepting the wife of the young man, who was then in the middle of her pregnancy, and who attended her husband during the few hours he survived the attack. A Maltese with a large family took a sick brother into his house, not suspecting that he laboured under the plague, and paid him unhesitatingly every affectionate attention. The instant this came to the knowledge of Mr. Iliff, apothecary to the forces, to whom the Maltese in question had formerly been a servant, the oil frictions were sent in and employed. The infected individual died, but the whole of the family escaped. It is needless to cite any other cases, although many similar ones might be adduced. The confidence of the Government of Malta in the efficacy of these frictions was at length so great, that a shed was erected at each of the barriers of Valetta, for the purpose of administering them to the guards stationed there, and to the market people and others whose avocations kept them abroad.

Alexis, the Piedmontese, who travelled every where in search of secrets, has published a variety of receipts for plague, into the composition of several of which storax enters. The writer was solicitous to make a trial of this substance, which unluckily was not to be found at Malta, either in the concrete state in which it is called storax *calamita*, or in its liquid state.* It was certainly, as the event proved, deserving such an essay, and was susceptible of various modifications in its use. What has been sanctioned by a long experience deserves credit, unless there be incontrovertible evidence to prove that the notion originally entertained of its efficacy was founded in error. A Turk, whose knowledge of the subject was by no means limited, distributed among his friends at Valetta lumps of a black substance resembling shoemaker's wax, which he had brought with him from Constantinople, and in the composition of which storax was the principal ingredient. These were either to be carried on a stick, and smelt to from time to time, or kept in the hand and constantly moulded, to the end, no doubt, that a portion of the substance might adhere to the fingers.

* It is with this latter substance that the fire-eaters, as they term themselves, anoint the tongue and fauces to protect them from the effect of caloric.

They

They were sought after with avidity. Now, it is to be observed that both the Seraglio cakes, and a particular description of beads of a great price in Turkey, are of a similar composition. It is, therefore, to be presumed, that those who brought them into use had something more in view than to supply an ornament in the case of the former; and in that of the latter, an object of pastime, in which light the generality of the Turks, who are in the constant habit of twirling them about with the fingers, regard beads.

Common tar, a bituminous substance which may be considered as in some degree analogous to storax, although it does not possess its peculiar fragrance, was employed by a Greek whose very hazardous task it was to bury the dead. With this substance he kept his hands and arms anointed. He was pointed out to the writer by Mr. Thomas, garrison surgeon, and acting superintendant of health, as having officiated with impunity in this way during the whole of the time that the plague raged; while the greater part of those who were similarly employed had been swept off by the contagion. In this instance it would appear, that the pores of the parts exposed to contact with the dead bodies were sheathed by the tar, so as to prevent the absorption of the plague matter; but in an old work entitled "The English Housewife," described by Beloe in his Anecdotes of Literature and Scarce Books, another application is made of that substance as a preservative against plague. It is recommended "to smell to a *nosegay* made of the tasselled end of a ship-rope," that is, of a tarred rope. Here something is implied of a specific quality of the tar, as a plague preventative; and this application of it agrees with that of the storax as employed by the Turks.—*Phil. Mag.*

### Mr. Hume's *improved Method of making Antimonium Tartar.*

Two parts of black *sulphuret of antimony* in fine powder, and one part of *nitrate of potash*, are to be mixed, and added to two parts of *sulphuric acid*, previously mixed with eight parts of water, and suffered to cool. By a due application of heat a proper oxide of antimony will be formed, which, when thoroughly washed, is to be boiled, while yet moist, with two parts of *supertartrate of potash* and a proper quantity of water. The solution is then to be filtrated, evaporated, and treated after the usual manner for crystallization.

Although I succeeded by other methods, peculiarly my own, to prepare this very important medicine, yet, upon the whole, I am disposed to prefer the formula which I now lay before you, more particularly as it is sanctioned with the decided approbation of those whom I wished to serve and consult.

I have here adopted the nomenclature of the College, which, after all, is as correct and convenient as any other that has been proposed for this article. I profess, indeed, to be an advocate for many of the arbitrary names, especially for such bodies as are beyond beyond a binary composition; and, where the ingredients enter, as in emetic tartar, alum, Rochelle salt, &c. in *definite* proportions, the rule is perfectly admissible; I should propose, therefore, to retain both these preparations of tartar, at least under their present titles, viz. *antimonium tartarisatum* and *soda tartarisata*.—*Phil. Mag.*

### CRITICAL ANALYSIS

